# Twelve weeks’ progressive resistance training combined with protein supplementation beyond habitual intakes increases upper leg lean tissue mass, muscle strength and extended gait speed in healthy older women

**DOI:** 10.1007/s10522-016-9671-7

**Published:** 2016-12-08

**Authors:** Peter Francis, William Mc Cormack, Clodagh Toomey, Catherine Norton, Jean Saunders, Emmet Kerin, Mark Lyons, Philip Jakeman

**Affiliations:** 1 Musculoskeletal Health Research Group, School of Clinical and Applied Science, Leeds Beckett University, Leeds, LS13HE UK; 2 Human Science Research Unit, Center for Intervention in Inflammation, Infection and Immunity, University of Limerick, Limerick, Ireland; 3 Food for Health Ireland, University of Limerick, Limerick, Ireland; 4 Statistical Consulting Unit/CSTAR @ UL, University of Limerick, Limerick, Ireland; 5 Sport Injury Prevention Research Centre, University of Calgary, Calgary, AB Canada

**Keywords:** Healthy aging, Sarcopenia, Muscle strength, Functional capability

## Abstract

The age-related decline in functional capability is preceded by a reduction in muscle quality. The purpose of this study was to assess the combined effects of progressive resistance training (PRT) and protein supplementation beyond habitual intakes on upper leg lean tissue mass (LTM), muscle quality and functional capability in healthy 50–70 years women. In a single-blinded, randomized, controlled design, 57 healthy older women (age 61.1 ± 5.1 years, 1.61 ± 0.65 m, 65.3 ± 15.3 kg) consumed 0.33 g/kg body mass of a milk-based protein matrix (PRO) for 12 weeks. Of the 57 women, 29 also engaged in a PRT intervention (PRO + PRT). In comparison to the PRO group (n = 28), those in the PRO + PRT group had an increase in upper leg LTM [0.04 (95% CI −0.07 to 0.01) kg vs. 0.13 (95% CI 0.08–0.18) kg, *P* = 0.027], as measured by Dual-energy X-ray absorptiometry; an increase in knee extensor (KE) torque [−1.6 (95% CI −7.3 to 4.4 N m) vs. 10.2 (95% CI 4.3–15.8 N m), *P* = 0.007], as measured from a maximal voluntary isometric contraction (Con-Trex MJ; CMV AG); and an increase in extended gait speed [-0.01 (95% CI −0.52–0.04) m s^−1^ vs. 0.10 (95% CI 0.05–0.22) m s^−1^, *P* = 0.001] as measured from a maximal 900 m effort. There was no difference between groups in the time taken to complete 5 chair rises or the number of chair rises performed in 30 s (*P* > 0.05). PRT in healthy older women ingesting a dietary protein supplement is an effective strategy to improve upper leg LTM, KE torque and extended gait speed in healthy older women.

## Introduction

Age associated alterations in skeletal muscle mass and architecture (Janssen et al. 2000; Thom et al. 2007), contractile properties (Miller and Toth 2013) and neuromuscular function (Brown 1972; Luff 1998) precede the age-related decline in functional capability Collectively, these components are thought to contribute to a muscles ‘quality’ and consequently there has been a growing interest in understanding the determinants of muscle performance rather than size. Expert working groups have recommended older adult muscle health is evaluated via the measurement of muscle mass, strength and functional capability (Cruz-Jentoft et al. 2010; Fielding et al. 2011). Expressing strength per unit lean tissue mass (LTM) provides an index of a muscles force generating capacity or muscle quality and has been reported to be a better predictor of functional capability in older adults than muscle or LTM alone (Hairi et al. 2010; Hayashida et al. 2014).

The primary (non-pharmacological) therapeutic interventions thought to increase muscle mass, strength and consequently functional capability are dietary and lifestyle interventions. A recent panel of experts (Bauer et al. 2013) have recommended that the recommended daily allowance (RDA) for dietary protein, currently 0.8 g kg^−1^ BW day^−1^, be increased to at least 1.0–1.2 g kg^−1^ BW day^−1^ in healthy older adults. This is thought necessary to offset the reported phenomenon of anabolic resistance to protein feeding that has been observed in the elderly (Breen and Phillips 2011; Paddon-Jones and Rasmussen 2009). Protein supplementation (0.165 g kg^−1^) at the two smaller meals of the day (normally breakfast and lunch) for a period of 6 months has been shown to increase LTM in healthy older adults (Norton et al. 2016).

It is well established, particularly in the young adult, that dietary protein and progressive resistance training act synergistically to promote the largest anabolic response (Breen and Phillips 2013) and subsequent increase in contractile mass (Cermak et al. 2012). Resistance training interventions of between 8 and 24 weeks have been shown to increase muscle mass, strength and functional capability in healthy older adults (Beneka et al. 2005; Eyigor et al. 2007; Rabelo et al. 2011; Tracy et al. 1999). However, there is a paucity of literature demonstrating the effects of resistance training in older adults who have had dietary protein optimised to at least the minimum required levels (1.0 g kg^−1^ BW day^−1^) The literature is particularly scant in relation to healthy older or middle aged (55–64 years; as defined by Glenn et al. 2014) women Therefore, the purpose of this study was to assess the effects of a PRT intervention on upper leg LTM, voluntary knee extensor (KE) torque, muscle quality (KE torque per unit upper leg LTM) and functional capability in healthy older women receiving dietary protein supplementation.

## Materials and methods

### Participants and design

A convenience sample (n = 204) of healthy older (50–70 years) adults was recruited via email and word of mouth from the University of Limerick campus community and surrounding area to take part in the University of Limerick Healthy Aging Study (Francis et al. 2016; Norton et al. 2016) Participants were screened by a medical doctor and provided a full medical history. Those defined as healthy, i.e. disease free based on Greig et al. (1994), independent-living, fully mobile and with no indication of dairy or lactose intolerance were invited to participate and to provide written informed consent. All participants were naïve to resistance training. All procedures were performed in accordance with the most recent version of the Declaration of Helsinki and approved by the research by the Faculty of Education and Health Sciences Research Ethics Committee (EHSREC 10/45). The trial was registered at clinicaltrials.gov as NCT02529124.

For the present investigation, 99 of the participants described above were female and were recruited to take part in a randomised, single-blinded, controlled trial with time (baseline compared with 12 wk) and treatment group [Protein Supplementation (PRO) or PRO + Progressive Resistance Training (PRO + PRT)] as independent factors. Simple randomisation was used to assign participants to a treatment group (PRO, n = 50 or PRO + PRT, n = 49). At the outset of the healthy ageing study whole body LTM was a principal outcome variable. Assuming a mean total LTM of 48 kg at entry to the study and an attainable mean change of ~1–2% (i.e. ~0.5–1 kg) over the period of the intervention, statistical power would be >80% at P < 0.05 for a study of 34 subjects. To undertake a study of women would therefore require 34 subjects per group with appropriately matched non-intervention controls. Six (6.1%) of participants were excluded following medical screening, 12 (12.1%) dropped out due to issues with supplement tolerance, 8 (8%) withdrew due to injury or illness, 7 (7.1%) dropped out due to time commitments, 4 (4%) were not compliant with the supplement, 2 (2%) dropped out due to family issues, 2 (2% dropped out due to relocation and 1 (1%) dropped out due to being unhappy with group allocation. The remaining 57 women had measures of dietary intake, whole and regional body composition, maximal voluntary isometric KE strength and functional capability taken prior to undergoing and after 12 weeks of treatment in the PRO (n = 28) or PRO + PRT (n = 29) groups.

**Fig. 1:**
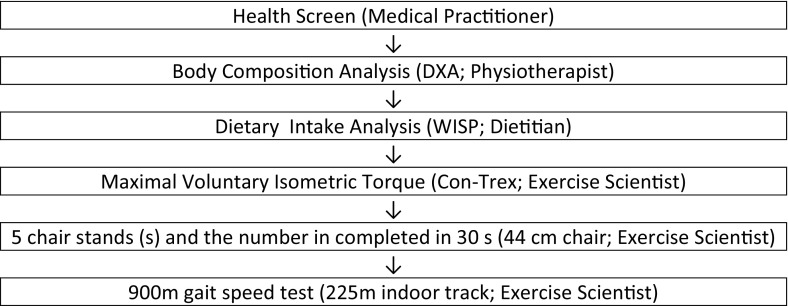
Order of study procedures, equipment used and personnel administering the procedure

Measurement of the dependent variables (Fig. 1) was normally conducted in the morning, often to facilitate fasted blood and body composition measurement. In a small number of instances participants who could not stay for the duration were offered afternoon testing slots. Participants were asked to refrain from caffeine intake that morning. Participants were asked to refrain from strenuous exercise 24 h before testing and to refrain from any exercise on the day of testing. Laboratory temperature was kept consistent at 22–24 °C and warm-up procedures were kept consistent for all measures. The only measure for which this was not controlled was the extended gait speed test which took place at a nearby public indoor running track. Post-testing was completed for all participants within 1 week of completing the exercise programme.

### Assessment of habitual dietary protein intake

The dietary analysis and regulation of supplement intake was undertaken by a registered dietician. Participants had habitual dietary intake assessed using a 4 days food diary encompassing two week days and two weekend days. The 4 days food diary was repeated twice separated by 7 days to ensure participants understood the detail required in their food records and to try and reduce issues with under-reporting. Food intake data were coded and subsequently analysed using WISP© (Tinuviel Software, Anglesey, UK) as previously described by Norton et al. (2016).

### Anthropometric and body composition analysis

Height was measured to the nearest 0.1 cm by using a stadiometer (Seca) and body mass (BM) was measured to the nearest 0.1 kg (MC-180MA; Tanita UK Ltd.). Whole body and regional body composition analysis was conducted using dual X-ray absorptiometry (iDXATM; GE Healthcare, Chalfont St Giles, Buckinghamshire, UK), in accordance with procedures used in the University of Limerick Body Composition Study (Leahy et al. 2013; Norton et al. 2016). The thigh, representing upper legLTM was measured from the inferior side of the lesser trochanter until the tibio-femoral joint.

### Maximal voluntary isometric contraction

Maximal voluntary isometric contractions of the knee extensors of the dominant limb were measured using a Con-Trex MJ Dynamometer (Con-Trex MJ; CMV AG, Dubendorf, Switzerland) in accordance with procedures used by Francis et al. (2016). Muscle quality was expressed as KE torque per kilogram upper leg LTM. This index has previously been reported as an appropriate index of muscle quality when using DXA and isokinetic dynamometry as the measurement tools (Francis et al. 2016).

### Functional capability

The ability to rise from a chair was assessed using a timed 5 repetition chair rise test (Guralnik et al. 1994) and by counting the number of chair rises completed in 30 s (Jones et al. 1999). Maximal extended gait speed, using a one or a combination of walking, jogging or running, was assessed by the time taken to complete 900 m (4 laps of an indoor track) (Francis et al., 2015). The outside lane of the track was measured as 225 m using a trundle wheel and all participants were asked to remain in the outside lane for the duration of the test. All baseline assessments of isometric strength and functional capability were repeated after a period of 7 days in order to reduce the potential for a learning effect. All measurements were carried out by the same exercise scientist to exclude issues with inter-tester reliability.

### Dietary protein supplementation

Participants were instructed to take a supplement at their two smaller protein containing meals of the day i.e. typically breakfast and lunch. Sachets were provided in powder format and could be mixed with water to make-up a powdered beverage or incorporated into meals. Supplements were prescribed relative to the median 4 levels of participants’ BM i.e. 45–59.9 kg, median of 52.5 kg; 60–74.9 kg, median of 67.5 kg; 75–89.9 kg, median of 82.5 kg and 90–105 kg, median of 97.5 kg). Each supplement dose provided 0.165 g protein kg^−1^ BW day^−1^ of median BM. Five flavours were available to off-set flavour fatigue and to improve compliance. A mixed excess number of supplement portions were provided every 4 weeks. with instructions that all used or unused sachets must be returned. Subjects were blinded to the supplement composition assigned to them. Compliance was monitored by count-back at the end of each month.

The milk protein matrix was composed of a 9:2:1 ratio of milk protein concentrate [80% (wt:wt protein); Glanbia Ingredients Ireland, Kilkenny Ireland]; whey protein concentrate hydrolysate, degree of hydrolysis 32% [WPC DH 32. 78% (wt:wt protein); Carbery Food Ingredients, Cork, Ireland; and whey protein isolate hydrolysate, degree of hydrolysis 45% [WPI DH 45, 75% (wt:wt protein); Glanbia Ingredients Ireland]. The total protein concentration was 72.7 g/100 g powder. The protein matrix was supplemented with 2187 mg/100 g powder of milk-based calcium (Trucal; Glanbia Ingredients Ireland) and 57.3 mg/100 g powder cholecalciferol. On the basis of 2 sachets/d, the daily supplement of milk protein matrix, milk-based calcium, and cholecalciferol was 0.33, 10, and 0.26 kg^−1^ median BM, respectively. A full breakdown of the supplement composition as recently been published (Norton et al. 2016).

### Progressive resistance training

Supervised (qualified sport and exercise scientist or chartered physiotherapist) exercise was performed on three non-consecutive days of week at a University sports hall. Each session lasted 45–60 min and consisted of a warm up, PRT and a cool down. Compliance to the exercise program was monitored using a weekly exercise log which was checked by the trainers administering the PRT session. The majority of sessions were conducted at 7:45 am to accommodate those with work commitments. Most participants would have consumed their dietary protein supplement within 2 h of the exercise session with breakfast. A smaller number of lunch-time and evening sessions were also provided for those who could not attend the early morning sessions. Participants were provided with equipment (aerobics step and therabands) and had the option of performing a maximum of 2 of the sessions at home, however most preferred to attend.

The first 3 weeks of the programme had an emphasis on ensuring the correct exercise technique and monitoring the appropriate amount of exercise and rest intervals for each individual. Between weeks 3 and 12, the programme was designed to promote muscle hypertrophy (4–6 sets, 8–15 repetitions) as recommended by Bird et al. (2005). The PRT programme consisted of a number of upper and lower body exercises using therabands (T) and body weight (BW) as the primary resistance. The primary exercises used throughout the program included squats (T), lunges (T), hip abduction (T), shoulder press (T), latissimus dorsi pull-down (or seated row) (T), bicep curls (T), calf raises (BW), push ups (BW), tricep dips (BW) and lumbopelvic stabilisation exercises. Theraband exercises were progressed by increasing the resistance of the band in ascending order: red, green, blue and black respectively. Push ups began against a wall; progressed to hands and knees and eventually assumed full push up position for some participants. Calf raises were completed as double legged exercises initially and then single whilst also varying the depth of ankle dorsi-flexion. Tricep dips were modified using step height (15, 20 and 25 cm). Progression was determined based on the participants post exercise rate of perceived exertion (RPE) in consultation with the exercise trainer. Exercises were performed with an emphasis on the eccentric phase of contraction. The eccentric phase was conducted to an audible count of 4 s, the position held for 1 s and the concentric phase completed in 2 s. Exercises were performed alternating between upper and lower body in order to minimize fatigue and optimize training time. Participants received 30 s rest between sets. The training programme was set out in 4 × 3 week progressive cycles (Cycle 1: 3 sets × 8 − 12 reps; Cycle 2: 3 sets × 14 reps; Cycle 3: 4 sets × 10 − 12 reps; Cycle 4: 4 sets × 12 − 14 reps).

### Statistical analysis

Baseline body-composition, muscle function and functional capability data were checked for normality of distribution by using the Shapiro–Wilk test and expressed as means ± SDs for normally distributed variables and medians (IQRs) for non-normally distributed variables. An independent t test or Mann–Whitney U test was used to compare differences between groups at baseline. The treatment effect was calculated as the change in outcome measure from baseline to 12 wk and presented as means or medians (95% CIs). These data were analysed by univariate ANOVA or Kruskal–Wallis Test with treatment (PRO compared with PRO + PRT) as a fixed factor. To determine the influence of baseline outcome measures on the changes seen at 12 wk, data were analysed by ANCOVA with group as a fixed factors and baseline value of the dependent variable as the covariate. Baseline dietary intake and compliance to the dietary protein supplement were also used as co-variates to determine their effects on changes in the dependent variables. Statistical analysis was performed by using PASW Statistics 22.0 for Windows (SPSS, Inc.). Significance (2-tailed) was set at P < 0.05 for all analyses.

## Results

Table 1 displays baseline participant characteristics, body composition, muscle function and functional capability for the 57 healthy older women who completed the study to 12 weeks. There were no differences in baseline measures between those in the PRO (n = 28) and the PRO + PRT (n = 29) groups (Table 1). Compliance to the dietary supplement was not different between the PRO and PRO + PRT groups respectively (86 ± 10% vs. 82 ± 10%, *P* = 0.213).

**Table 1:** Baseline participant characteristics, body composition, muscle function and functional capability in healthy older women

	PRO (n = 28)	PRO + PRT (n = 29)	*P* ^a^
Age (years)	61.8 ± 4.5	60.4 ± 5.6	0.324
Height (cm)	160.4 (7.8)	162.0 (6.0)	0.637
Body mass (kg)	68.6 ± 12.2	64.9 (10.9)	0.666
BMI (kg m^−2^)	26.1 ± 3.9	24.7 (4.1)	0.439
Body fat (%)	37.7 ± 6.6	36.7 ± 5.0	0.515
LTM (kg)	39.9 ± 4.8	39.9 ± 3.5	0.811
Protein intake (g kg day^−1^)	1.22 ± 0.34	1.29 ± 0.34	0.432
Upper limb LTM (kg)	3.68 ± 0.60	3.71 ± 0.52	0.865
Knee extensor peak torque (N m)	89.0 (26.5)	86.2 ± 22.8	0.346
Muscle quality (N m kg^−1^/LTM)	25.7 ± 6.5	23.3 ± 5.8	0.153
900 m gait speed (m s^−1^)	2.14 (0.50)	2.29 ± 0.47	0.587
5 Chair repetition (s)	8.3 ± 2.0	8.4 (2.4)	0.696
Chair repetitions in 30 s (n)^b^	18 ± 5	18 ± 4	0.826

Values are means ± SDs or medians (IQR). No significant differences were found between groups

^a^P values for the difference between PRO and PRO + EX groups analysed by independent t test or Mann–Whitney U test

^b^PRO, n = 17, PRO + PRT, n = 14

**Fig. 2:**
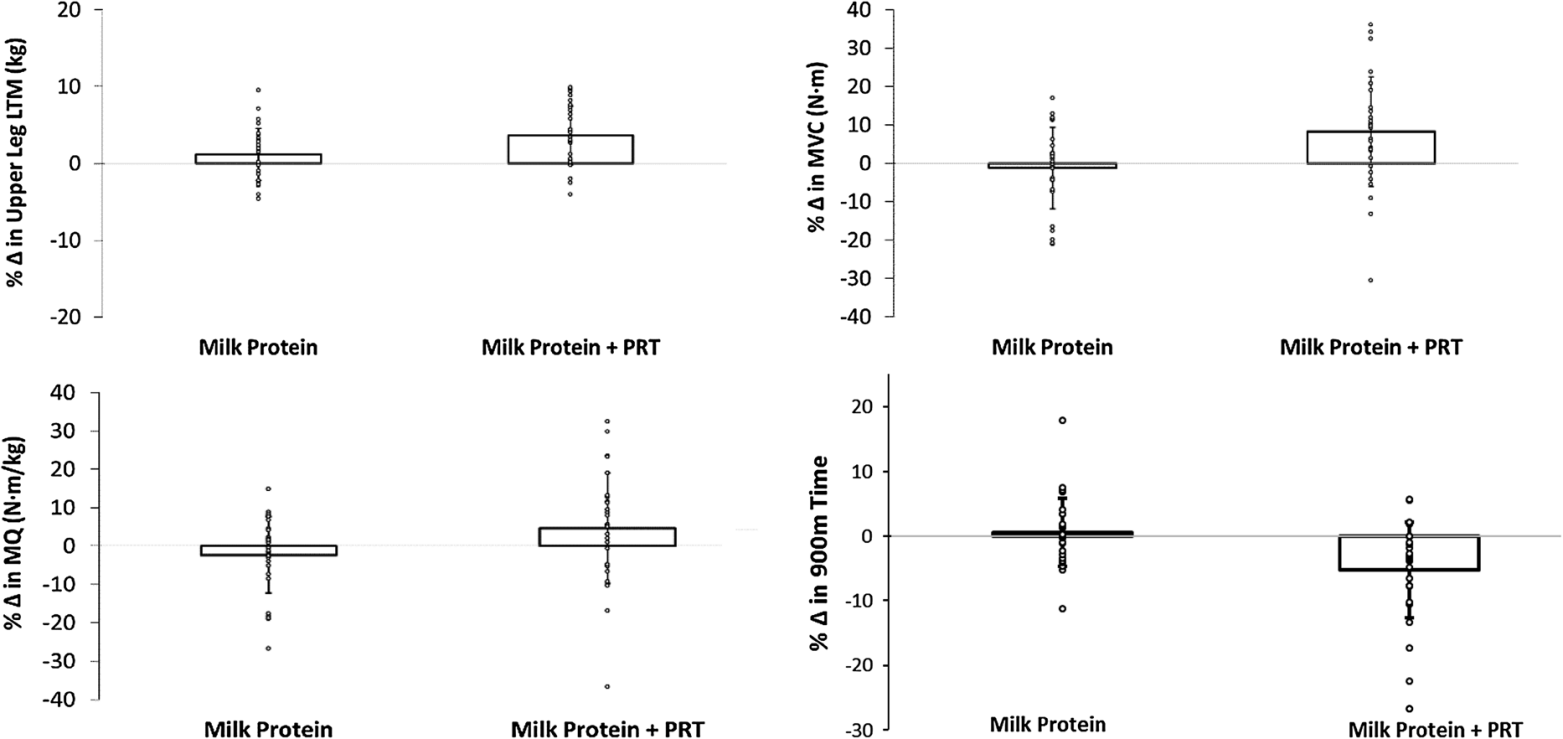
Mean and individual relative percentage change in dependent variables

There was a net positive change in upper leg LTM in both the PRO and PRO + EX groups although greater in the PRO + EX group (3.6% vs. 1.2%, *P* = 0.017). Muscle function as assessed by KE torque and KE torque per kg upper leg LTM (muscle quality) demonstrated positive relative gains in the PRO + EX group but were negative in the PRO group. Extended gait speed demonstrated positive absolute and relative gains in the PRO + EX group but remained unchanged in the PRO group (Fig. 2). The time taken to complete 5 chair rises and the number of chair rises complete in 30 s remained unchanged in both groups (Table 2).

**Table 2:** Change in LTM, muscle function and functional capability in healthy older women

	Treatment effect			Treatment effect (%)		
	**∆**	*P* ^a^	1 − β	∆	*P* ^a^	1 − β
LTM (kg)						
PRO	−0.21 (−0.8 to 0.3)	0.133		−0.6 (−2.0 to 0.9)	0.142	
PRO + EX	0.34 (−0.2 to 0.9)			1.0 (−0.5 to 2.5)		
Upper leg LTM (kg)						
PRO	0.04 (−0.07 to 0.01)	0.027	0.606	1.2 (−0.2 to 2.6)	0.017	0.680
PRO + EX	0.13 (0.08–0.18)			3.6 (2.3 to 5.0)		
Knee extensor torque (N m)						
PRO	−1.6 (−7.3 to 4.4)	0.007	0.784	−1.3 (−7.8 to 5.9)	0.008	0.766
PRO + EX	10.2 (4.3–15.8)			12.7 (5.6–19.0)		
Muscle quality (N m kg^−1^)						
PRO	−0.8 (−2.2 to 1.0)	0.062		−2.4 (−8.1 to 5.1)	0.046	0.520
PRO + EX	1.8 (0.0 to 3.2)			9.0 (1.6–14.6)		
900 m gait speed (m s^−1^)^b^						
PRO	−0.01 (−0.52 to.−0.04)	0.001		−0.4 (−2.6 to 1.8)	0.001	
PRO + EX*	0.10 (0.05–0.22)			3.8 (3.0–8.4)		
5 Chair REP (s)^b^						
RO	−0.01 (−0.48 to 0.45)	0.749		0.8 (−4.9 to 6.5)	0.793	
PRO + EX*	0.01 (−0.23 to 0.60)			0.1 (−2.8 to 6.8)		
Chair REP 30 s (n)^c^						
PRO	−1.0 (−1.8 to −0.2)	0.572		−4.2 (−7.8 to −6.2)	0.904	
PRO + EX*	−0.5 (−2.0 to 0.0)			−5.3 (−10.0 to 0.0)		

*LTM* lean tissue mass, *REP* repetition, 1 − β statistical power

Data are means (95% CIs) and * medians (Bootstrap 95% CIs). Treatment effect = (value at 12 week − value at baseline)

^a^P values for the difference in treatment effect, PRO compared with PRO + PRT, analysed by univariate ANOVA or Kruskall Wallis Test with treatment group as a fixed factor

^b^PRO, n = 25, PRO + PRT, n = 26

^c^PRO, n = 17, PRO + PRT, n = 14

Baseline outcome measures, dietary protein intake at baseline and compliance to the dietary protein supplement did not have an effect on changes in the dependent variables when controlled for as covariates in the analysis (*P* > 0.05). Relative changes in upper leg LTM were not associated with relative changes in extended gait speed. Relative changes in KE torque corrected for body mass had the strongest association with relative change in extended gait speed followed by muscle quality, although both these correlations were of moderate strength (Table 3).

**Table 3:** Pearson correlations between relative change in laboratory measures of muscle function and relative change in 900 m performance

∆ % Laboratory measures	∆ % Gait speed (m/s)^a^	P*
Upper leg LTM (kg)	0.005	0.972
KE torque/BM (N m kg^−1^)	0.514	<0.001
Muscle quality (N m kg^−1^)	0.478	<0.001

^a^Pearsons r

* *P* values for the correlations reported

## Discussion

Ingestion of dietary protein and PRT promote a synergistic acute anabolic response in healthy adults (Snijders et al. 2015). Therefore, it would seem plausible that this strategy would promote the largest gains in contractile mass and subsequent improvement in muscle function when extended over a 12-week period. To our knowledge this is the first randomised controlled trial to investigate the effects of PRT on LTM, muscle function and functional capability in healthy older women supplemented with dietary protein to ensure an absolute intake of >1.0 g kg^−1^ BW day^−1^. Compared to the PRO group we demonstrate greater relative gains in upper leg LTM, KE torque and muscle quality in the PRO + PRT group. Functional capability as measured by extended gait speed demonstrated greater absolute and relative gains in the PRO + PRT group compared to the PRO group. Functional capability as measured by short (5 chair rises) or extended (number of chair rises in 30 s) chair rise tests remained unchanged in both groups. Relative changes in KE torque corrected for upper leg LTM or body mass had a moderate association with relative changes in extended gait speed.

Whole body LTM remained unchanged between groups after the 12-week intervention, whilst upper leg LTM demonstrated a modest 3.6% (~130 g) increase in the PRO + EX group. In light of the measurement error (2.3%) for upper leg LTM using DXA this difference may be considered negligible. However, as 95% confidence intervals do not cross zero in the PRO + PRT group, it is more likely that a true difference does exist and that DXA overestimates baseline skeletal muscle mass and underestimate changes in response to PRT (Delmonico et al. 2007; Maden-Wilkinson et al. 2013). This is largely due to DXA’s inability to differentiate between skin, skeletal muscle and other fat-free tissue components (Wang et al. 1996). This methodology may mask changes in LTM at the whole body level and underestimate changes at the upper leg. The fact that DXA underestimates skeletal muscle change during interventions is perhaps also the first step towards explaining the 3.5 times greater increase in KE torque compared with upper leg LTM in the PRO + PRT group. In fact, the ratio of age-related difference in upper leg LTM and KE torque (1:3) previously reported in this population (Francis et al. 2016) appears to mirror the ratio of intervention led improvement in the present investigation. While the work of Häkkinen et al. (2001) demonstrates a closer approximation of increases in mass (5.5%) and KE torque (8.7%) when using computed tomography (CT) to measure responses to 12 weeks PRT in women (~74 years), it is unlikely that DXA underestimates threefold increases in upper leg skeletal muscle mass.

The magnitude of KE torque increase is lightly to due to improvements in muscle quality. The increase in muscle quality is likely due to a reversal of some of the adverse physiological changes associated with muscle ageing such as a reduction in the angle of muscle pennation (Thom et al. 2007), the number and diameter of type I and type IIA muscle fibres (Lexell et al. 1988; Nilwik et al. 2013) and the number of active motor units (Luff 1998), all of which, are compounded by an increase in connective tissue and fat infiltration (Taaffe et al. 2009). These changes in muscle quality reduce the contractile mass and subsequently the force generating capacity of the muscle. Participants in this study were naïve to PRT. The improvement in muscle quality and functional capability may also in part be due to a normal response to exercise training separate to that of reversing adverse changes associated ageing. For example, at the cellular level, the mitochondrial response to exercise training is an intact process in aging skeletal muscle and this may contribute to the beneficial effects of exercise on musculoskeletal health with aging (Cobley et al. 2015). Furthermore, at the whole body level, cessation of exercise in trained older adults increases the fatty infiltration and reduces strength of the muscle while resumption has the opposite effect (Taaffe et al. 2009). It is perhaps the neurological aspects of muscle quality which are restored initially by PRT in healthy older or middle aged adults; accompanied by only modest increases in muscle mass. The basis for this suggestion comes from the fact that we did not observe the absolute (0.5–0.7 kg) nor relative (1.2–1.9%) improvements in whole body LTM reported from women undergoing PRT in previous literature (Rabelo et al. 2011; Tracy et al. 1999). One possible explanation for this is that these studies have tended to investigate women greater than 60 years often with a mean age of ~70 years. The fact that strength improvements in these studies are similar to ours but LTM gains are greater may suggest that muscle mass and function are more closely linked in older adults >60 years. Support for this suggestion comes from the work of Newman et al. (2003) who reports an 8.6% per decade decline in leg LTM and an accompanying 10.1% change in muscle quality. The proximity between changes in muscle mass and strength in older adults suggests there is a threshold, after which, changes in muscle quality which began earlier in life (~45 years) are now accompanied by declines in skeletal muscle mass.

The findings of an improvement in KE peak torque and subsequent improvement in muscle quality is in agreement with previous literature which has reported older women to have a 7–21% (6–13 N m) increase in KE peak torque from interventions lasting between 8 and 24 weeks (Beneka et al. 2005; Damush and Damush 1999; Rabelo et al. 2011; Tracy et al. 1999). This relatively narrow range of improvements in spite of the varying duration and modes of resistance exercise employed suggests that muscles worked close to their force generating capacity will get stronger (Morton et al. 2016). This suggestion is underpinned by the work of Häkkinen et al. (2001) who reported activation of the largest motor units to occur after the first 7 weeks of resistance training. Our results also support existing data which suggest that age-related difference (Janssen et al. 2000; Lynch et al. 1999) and intervention led improvements (Häkkinen et al. 2001) in skeletal muscle mass are greatest at the thigh region.

The identification of laboratory measures of LTM and muscle function which can detect age-related difference and are sensitive to therapeutic intervention become clinically relevant when associated with functional capability. We have previously reported the chair rise and extended gait speed tests used in this study as capable of detecting age-related difference in healthy 50–70 years women Francis et al. 2015). Furthermore, all measures were associated with maximal KE torque. In the present investigation the short (5 repetitions) and extended (number complete in 30 s) chair rise tests were not different between the PRO and PRO + EX after 12 weeks. Therefore, it would appear that while chair rise tests can detect age-related difference in functional capability between decades (6th and 7th) they do not have the sensitivity to detect changes as a result of short term therapeutic intervention. The improvement in extended gait speed for those in the PRO + EX group, practically speaking, was equivalent to a 20 s reduction in the time taken to complete 900 m. This test may demonstrate greater sensitivity due to enabling participants to perform to a greater maximum pre and post intervention. Tests of short duration (<30 s) may suffer from a ceiling effect when used in short term interventions with healthy older adults. Relative improvements in extended gait speed were associated with relative improvement in KE peak torque and muscle quality but not upperleg LTM. These findings are consistent with our cross-sectional work which reported associations between upper leg LTM, KE torque, muscle quality and measures of functional capability in a larger (n = 128) sample of healthy 50–70 years (Francis 2014). Interestingly, compared to muscle quality, KE torque corrected for body mass had a consistently stronger association with all measures of functional capability. The association although highly statistically significant is still moderate (r = 0.514, *P* = <0.001) which is most likely due to the number of other factors which influence the time taken to complete 900 m. It is likely that an increase in capillary angiogenesis and mitochondrial biogenesis would occur in participants exposed to a new training stimulus involving local muscular endurance, improving delivery and extraction of oxygen by working muscles (Holloszy and Coyle 1984). These findings support those of Visser et al. (2005), Hairi et al. (2010) and Hayashida et al. (2014) who have consistently identified muscle strength rather than LTM as a key determinant of functional capability in older adults >65 years. There are two potentially important findings from this aspect of the work (a) KE torque corrected for body mass may be as relevant to the performance of functional tasks as muscle quality; most likely due to the fact that it represents strength relative to the body mass being accelerated and (b) extended gait speed tests; which allow performance to a greater maximum than short test batteries may be required to detect changes in short term interventions using healthy older women. The basis for this suggestion comes from data reported by Glenn et al. (2014) which indicated no difference in 10 m habitual gait speed between older adults who are sedentary, recreationally active or masters athletes and no difference in 10 m maximal gait speed between those who are sedentary or recreationally active. We previously reported extended gait speed, as used in this study, but not short (10 m) gait speed to demonstrate age-related difference between the 6th and 7th decade of life (Francis et al. 2015). These findings combined with the findings in the present study suggest healthy older adults need to be challenged sufficiently in order to establish meaningful degradations in capacity and/or response to intervention.

The fact that baseline dietary protein intake (P = 0.596) or supplement compliance (P = 0.286) had no effect on change in upperleg LTM is perhaps due to the fact that the mean baseline intake for the intervention cohort (n = 57) was 1.22 g protein·kg^−1^ BW day^−1^ prior to supplementation. An expert working group (Bauer et al. 2013) suggested an intake of 1.0–1.2 g protein kg^−1^ BW day^−1^ was required to overcome the anabolic resistance identified in aging muscle. It may be that current intakes in this convenience sample of 50–70 years old women were already optimised. Our results are in agreement with that of Andrews et al. (2006) in men and women, Kukuljan et al. (2009) in men and Chalé et al. (2013) in mobility limited older adults who have reported protein intake at baseline and whey protein supplementation not to have significant effect on changes in LTM as a result of PRT. Macnaughton et al. (2016) have demonstrated that 40 g of protein supplementation post whole body resistance training increases 24 h MPS to a greater extent than 20 g in healthy young men. Our protein dose post exercise (normally breakfast) was ~8 to 16 g dependent on BM and ~16 to 32 g per day when both supplements are included. Although this would have led to an even distribution of protein (~ 30 g protein/meal) throughout the day it may not have been sufficient to optimise MPS post exercise, particularly in older adults which may require additional protein to overcome anabolic resistance. Furthermore, although we carefully determined baseline dietary protein intake at baseline so that we could supplement to optimise the daily dose; we did not monitor dietary protein intake throughout the intervention and therefore cannot be sure dietary protein intake remained adequate throughout the intervention. There was a high drop-out rate in our study and almost half of it came from issues related to supplement tolerance and compliance. This is despite the availability of 5 flavors to off-set flavor fatigue and to improve compliance. Researchers may want to consider which mode of protein delivery might maximise compliance. 22% of participants dropped out due to injury or illness (not study related) and 19% due to time commitments. A consideration for future research of this nature is the time cost required from participants, particularly those who are still in full-time employment. This is largely influenced by the number of dependent or control variables being measured during the intervention period and may have influenced dropout rate. In summary, we report positive gains in upper leg LTM, KE torque and muscle quality for those undergoing 12 weeks of PRT in addition to protein supplementation beyond habitual intakes. Extended gait speed, as assessed by a maximal 900 m effort, was the only measure of functional capability capable of detecting change as a result of a PRT intervention. Improvements in extended gait speed were associated with improvements in KE strength and muscle quality but not upper leg LTM. Future studies in healthy older adults should consider optimising the protein dose, habitual distribution and post exercise intake to see if LTM and muscle function responses can be augmented post PRT interventions.

## References

[CR1] Andrews RD, MacLean DA, Riechman SE (2006). Protein intake for skeletal muscle hypertrophy with resistance training in seniors. Int J Sport Nutr Exerc Metab.

[CR2] Bauer J (2013). Evidence-based recommendations for optimal dietary protein intake in older people: a position paper from the PROT-AGE study group. J Am Med Dir Assoc.

[CR3] Beneka A, Malliou P, Fatouros I, Jamurtas A, Gioftsidou A, Godolias G, Taxildaris K (2005). Resistance training effects on muscular strength of elderly are related to intensity and gender. J Sci Med Sport.

[CR4] Bird SP, Tarpenning KM, Marino FE (2005). Designing resistance training programmes to enhance muscular fitness. Sports Med.

[CR5] Breen L, Phillips S (2011). Skeletal muscle protein metabolism in the elderly: interventions to counteract the ‘anabolic resistance’ of ageing. Nutr Metab.

[CR6] Breen L, Phillips SM (2013). Interactions between exercise and nutrition to prevent muscle waste during ageing. Br J Clin Pharmacol.

[CR7] Brown WF (1972). A method for estimating the number of motor units in thenar muscles and the changes in motor unit count with ageing. J Neurol Neurosurg Psychiatry.

[CR8] Cermak NM, Res PT, de Groot LC, Saris WH, van Loon LJ (2012). Protein supplementation augments the adaptive response of skeletal muscle to resistance-type exercise training: a meta-analysis. Am J Clin Nutr.

[CR9] Chalé A, Cloutier GJ, Hau C, Phillips EM, Dallal GE, Fielding RA (2013). Efficacy of whey protein supplementation on resistance exercise-induced changes in lean mass, muscle strength, and physical function in mobility-limited older adults. J Gerontol Ser A.

[CR10] Cobley JN, Moult PR, Burniston JG, Morton JP, Close GL (2015). Exercise improves mitochondrial and redox-regulated stress responses in the elderly: better late than never!. Biogerontology.

[CR11] Cruz-Jentoft AJ (2010). Sarcopenia: European consensus on definition and diagnosis: Report of the European Working Group on Sarcopenia in Older People. Age Ageing.

[CR12] Damush TM, Damush JG (1999). The effects of strength training on strength and health-related quality of life in older adult women. Gerontologist.

[CR13] Delmonico M, Kostek M, Johns J, Hurley B, Conway J (2007). Can dual energy X-ray absorptiometry provide a valid assessment of changes in thigh muscle mass with strength training in older adults&quest. Eur J Clin Nutr.

[CR14] Eyigor S, Karapolat H, Durmaz B (2007). Effects of a group-based exercise program on the physical performance, muscle strength and quality of life in older women. Arch Gerontol Geriatr.

[CR15] Fielding RA (2011). Sarcopenia: an undiagnosed condition in older adults. Current consensus definition: prevalence, etiology, and consequences. International working group on sarcopenia. J Am Med Dir Assoc.

[CR16] Francis P (2014) Age-related change in muscle mass, strength and function in healthy adult Irish women. PhD Thesis submitted to the University of Limerick. Available at: https://ulir.ul.ie/handle/10344/4255

[CR17] Francis P, Mc Cormack W, Lyons M and Jakeman P (2015) Measurement of the ability to perform activities related to daily living in healthy older 50–70 years adults. Proceedings of the active healthy ageing, sports science and neuroscience conference, Magdeburg, Germany, 2–5 September 2015

[CR18] Francis P, Toomey C, Mc Cormack W, Lyons M, Jakeman P (2016). Measurement of maximal isometric torque and muscle quality of the knee extensors and flexors in healthy 50- to 70-year-old women. Clin Physiol Funct Imaging.

[CR19] Greig CA, Young A, Skelton DA, Pippet E, Butler FMM, Mahmud SM (1994). Exercise studies with elderly volunteers. Age and Ageing.

[CR20] Glenn J, Vincenzo J, Canella C, Binns A, Gray M (2014). Habitual and maximal dual-task gait speeds among sedentary, recreationally active, and masters athlete late-middle aged adults. J Aging Phys Act.

[CR21] Guralnik JM (1994). A short physical performance battery assessing lower extremity function: association with self-reported disability and prediction of mortality and nursing home admission. J Gerontol.

[CR22] Hairi NN (2010). Loss of muscle strength, mass (sarcopenia), and quality (specific force) and its relationship with functional limitation and physical disability: the Concord Health and Ageing in Men Project. J Am Geriatr Soc.

[CR23] Häkkinen K, Pakarinen A, Kraemer WJ, Häkkinen A, Valkeinen H, Alen M (2001). Selective muscle hypertrophy, changes in EMG and force, and serum hormones during strength training in older women. J Appl Physiol.

[CR24] Hayashida I, Tanimoto Y, Takahashi Y, Kusabiraki T, Tamaki J (2014). Correlation between muscle strength and muscle mass, and their association with walking speed, in community-dwelling elderly Japanese individuals. PLoS ONE.

[CR25] Holloszy JO, Coyle EF (1984). Adaptations of skeletal muscle to endurance exercise and their metabolic consequences. J Appl Physiol.

[CR26] Janssen I, Heymsfield SB, Wang Z, Ross R (2000). Skeletal muscle mass and distribution in 468 men and women aged 18–88 year. J Appl Physiol.

[CR27] Jones CJ, Rikli RE, Beam WC (1999). A 30-s chair-stand test as a measure of lower body strength in community-residing older adults. Res Q Exerc Sport.

[CR28] Kukuljan S, Nowson CA, Sanders K, Daly RM (2009). Effects of resistance exercise and fortified milk on skeletal muscle mass, muscle size, and functional performance in middle-aged and older men: an 18-mo randomized controlled trial. J Appl Physiol.

[CR29] Leahy S, O’Neill C, Sohun R, Toomey C, Jakeman P (2013). Generalised equations for the prediction of percentage body fat by anthropometry in adult men and women aged 18–81 years. Br J Nutr.

[CR30] Lexell J, Taylor CC, Sjostrom M (1988). What is the cause of the ageing atrophy? Total number, size and proportion of different fiber types studied in whole vastus lateralis muscle from 15- to 83-year-old men. J Neurol Sci.

[CR31] Luff AR (1998). Age-associated changes in the innervation of muscle fibers and changes in the mechanical properties of motor units. Ann N Y Acad Sci.

[CR32] Lynch N (1999). Muscle quality. I. Age-associated differences between arm and leg muscle groups. J Appl Physiol.

[CR33] Macnaughton LS (2016). The response of muscle protein synthesis following whole‐body resistance exercise is greater following 40 g than 20 g of ingested whey protein. Physiol Rep.

[CR34] Maden-Wilkinson T, Degens H, Jones D, McPhee J (2013). Comparison of MRI and DXA to measure muscle size and age-related atrophy in thigh muscles. J Musculoskelet Neuronal Interact.

[CR35] Miller MS, Toth MJ (2013). Myofilament protein alterations promote physical disability in aging and disease. Exerc Sport Sci Rev.

[CR36] Morton RW (2016). Neither load nor systemic hormones determine resistance training-mediated hypertrophy or strength gains in resistance-trained young men. J Appl Physiol.

[CR37] Newman AB (2003). Strength and muscle quality in a well-functioning cohort of older adults: the health aging and body composition study. J Am Geriatr Soc.

[CR38] Nilwik R, Snijders T, Leenders M, Groen BB, van Kranenburg J, Verdijk LB, van Loon LJ (2013). The decline in skeletal muscle mass with aging is mainly attributed to a reduction in type II muscle fiber size. Exp Gerontol.

[CR39] Norton C, Toomey C, McCormack WG, Francis P, Saunders J, Kerin E, Jakeman P (2016). Protein supplementation at breakfast and lunch for 24 weeks beyond habitual intakes increases whole-body lean tissue mass in healthy older adults. J Nutr.

[CR40] Paddon-Jones D, Rasmussen B (2009). Dietary protein recommendations and the prevention of sarcopenia. Curr Opin Clin Nutr Metab Care.

[CR41] Rabelo HT, Bezerra LA, Terra DF, Lima RM, Silva MA, Leite TK, de Oliveira RJ (2011). Effects of 24 weeks of progressive resistance training on knee extensors peak torque and fat-free mass in older women. J Strength Cond Res.

[CR42] Snijders T (2015). Protein ingestion before sleep increases muscle mass and strength gains during prolonged resistance-type exercise training in healthy young men. J Nutr.

[CR43] Taaffe DR, Henwood TR, Nalls MA, Walker DG, Lang TF, Harris TB (2009). Alterations in muscle attenuation following detraining and retraining in resistance-trained older adults. Gerontology.

[CR44] Thom J, Morse C, Birch K, Narici M (2007). Influence of muscle architecture on the torque and power–velocity characteristics of young and elderly men. Eur J Appl Physiol.

[CR45] Tracy B (1999). Muscle quality. II. Effects of strength training in 65- to 75-year-old men and women. J Appl Physiol.

[CR46] Visser M (2005). Muscle mass, muscle strength, and muscle fat infiltration as predictors of incident mobility limitations in well-functioning older persons. J Gerontol Ser A.

[CR47] Wang Z, Visser M, Ma R, Baumgartner R, Kotler D, Gallagher D, Heymsfield S (1996). Skeletal muscle mass: evaluation of neutron activation and dual-energy X-ray absorptiometry methods. J Appl Physiol.

